# Comparing intentions to reduce substance use and willingness to seek help among transgender and cisgender participants from the global drug survey

**DOI:** 10.1192/bjo.2021.654

**Published:** 2021-06-18

**Authors:** Dean Connolly, Emma Davies, Michael Lynskey, Monica Barratt, Larissa Maier, Jason Ferris, Adam Winstock, Gail Gilchrist

**Affiliations:** 1 IoPNN King's College London; 2 Oxford Brookes University; 3 IoPPN King's College London; 4 RMIT University; 5 University of California; 6 The University of Queensland; 7 University College London

## Abstract

**Aims:**

To describe and compare psychoactive substance misuse help-seeking among transgender (trans) and cisgender (cis) participants from a large multi-national cross-sectional survey.

**Background:**

Trans people experience stressors related to their minority status which have been associated with increased rates of psychoactive substance use and related harm. Despite this, there is a paucity of evidence relating to the treatment needs of trans people who use psychoactive substances, beyond a small body of literature describing a culture of transphobic hostility in general substance misuse services. This paper aims to describe and compare psychoactive substance misuse help-seeking among trans and cis participants from a large multi-national cross-sectional survey.

**Method:**

Over 180,000 participants, recruited from the world's largest annual survey of drug use - the Global Drug Survey (GDS) - during 2018 and 2019, reported use of a range of psychoactive substances in the preceding 12 months. Five gender groups (118,157 cis men, 64,319 cis women, 369 trans men, 353 trans women and 1,857 non-binary people) were compared, using Chi-square and z-tests with Bonferroni correction, on items relating to the desire to use less psychoactive substances and the need to seek help to achieve this. Respondents from GDS 2018 were also assessed for substance dependence. Binary logistic regression was used to compare gender groups on self-reported substance dependence to frame the help-seeking analyses.

**Result:**

Trans respondents (n = 1,710) to GDS 2018 were significantly more likely than cis respondents to report use of illicit substances (OR = 1.66-2.93) and dependence on cannabis (OR = 2.39), alcohol (OR = 3.28) and novel psychoactive substances (OR = 4.60). In the combined GDS 2018 and 2019 dataset, there were no significant differences between trans (n = 2,579) and cis (n = 182,476) participants on the desire to reduce substance use. However, among those who did report wanting to use less, non-binary people and trans women were most likely to want help to achieve this.

**Conclusion:**

Trans respondents reported a greater need for help with reducing substance use than cis respondents. Given the deficit of specialist services for psychoactive substance users who are trans, there is a need for a more thorough understanding of the barriers and facilitators to their engagement in general substance misuse services. In the interim, substance misuse service providers require education about gender minority status to help meet the needs of trans clients.

